# Cortical Gray Matter and Hippocampal Atrophy in Idiopathic Rapid Eye Movement Sleep Behavior Disorder

**DOI:** 10.3389/fneur.2019.00312

**Published:** 2019-04-05

**Authors:** Anna Campabadal, Barbara Segura, Carme Junque, Monica Serradell, Alexandra Abos, Carme Uribe, Hugo C. Baggio, Carles Gaig, Joan Santamaria, Yaroslau Compta, Nuria Bargallo, Alex Iranzo

**Affiliations:** ^1^Medical Psychology Unit, Department of Medicine, Institute of Neuroscience, University of Barcelona, Barcelona, Spain; ^2^Centro de Investigación Biomédica en Red sobre Enfermedades Neurodegenerativas (CIBERNED), Hospital Clínic de Barcelona, Barcelona, Spain; ^3^Neuropsychology Group, Clinical and Experimental Neuroscience, Institute of Biomedical Research August Pi i Sunyer, Barcelona, Spain; ^4^Multidisciplinary Sleep Unit, Hospital Clínic, Barcelona, Spain; ^5^Movement Disorders Unit, Neurology Service, Institute of Neuroscience, Hospital Clínic de Barcelona, University of Barcelona, Barcelona, Spain; ^6^Centre de Diagnòstic per la Imatge, Hospital Clínic, Barcelona, Spain

**Keywords:** idiopathic REM-sleep behavior disorder, MRI-magnetic resonance imaging, cortical gray matter atrophy, hippocampal atrophy, hippocampal subfields, cognition

## Abstract

**Objective:** In this study we investigate cortical and subcortical gray matter structure in patients with Idiopathic REM-sleep behavior disorder (IRBD), and their relation to cognitive performance.

**Methods:** This study includes a sample of 20 patients with polysomnography-confirmed IRBD and 27 healthy controls that underwent neuropsychological and T1-weighted MRI assessment. FreeSurfer was used to estimate cortical thickness, subcortical volumetry (version 5.1), and hippocampal subfields segmentation (version 6.0). FIRST, FSL's model-based segmentation/registration tool was used for hippocampal shape analysis.

**Results:** Compared with healthy subjects, IRBD patients showed impairment in facial recognition, verbal memory, processing speed, attention, and verbal naming. IRBD patients had cortical thinning in left superior parietal, post-central, and fusiform regions, as well as in right superior frontal and lateral occipital regions. Volumetric and shape analyses found right hippocampal atrophy in IRBD, specifically in posterior regions. Hippocampal subfields exploratory analysis identified significant differences in the right CA1, molecular layer, granule cell layer of dentate gyrus, and CA4 of this patients. No correlations were found between cognitive performance and brain atrophy.

**Conclusion:** This work confirms the presence of posterior based cognitive dysfunction, as well as cortical and right hippocampal atrophy in IRBD patients.

## Introduction

Rapid eye movement (REM) sleep behavior disorder (RBD) is a parasomnia characterized by abnormal motor and vocal behaviors associated with unpleasant dreams and increased electromyographic activity during REM sleep (1). Polysomnography with audiovisual recording is needed to confirm the diagnosis of RBD and to exclude other sleep disorders that can mimic its symptoms, including obstructive sleep apnea, nocturnal hallucinations, and confusional arousals (2).

Over the past years, the idiopathic form of RBD (IRBD) has been increasingly recognized as a prodromal phase of some neurodegenerative diseases, mainly of alpha-synucleinopathies such as dementia with Lewy bodies (DLB), Parkinson's disease (PD), and multiple system atrophy (3, 4). The risk for IRBD patients of eventually developing a neurodegenerative disease increases with time (3, 5). IRBD is by far the strongest and most specific clinical predictor of neurodegenerative disease available (6). Therefore, there is growing interest in describing neuroimaging and cognitive biomarkers of brain neurodegeneration in this prodromal disorder.

With an estimated prevalence of 50%, cognitive impairment is frequently present in IRBD patients (7), affecting manly attention, verbal memory, visuospatial, and executive domains (8). Furthermore, previous works demonstrated cognitive decline in attention and executive functions strongly predict conversion to DLB in IRBD patients (9).

Structural brain imaging techniques have been used to investigate neurodegenerative changes in IRBD. Previous works using diffusion-tensor imaging showed that IRBD patients had decreased fractional anisotropy in the tegmentum of the midbrain, increased mean diffusivity in the pontine reticular formation (10), and microstructural changes in widespread areas including brainstem, substantia nigra, temporal lobe, and visual stream (11). Voxel-based morphometry (VBM) studies revealed that IRBD patients had gray matter volume reduction in the superior frontal sulcus (12), anterior cingulate gyrus, caudate nucleus (13), anterior lobes of the cerebellum, tegmental portion of the pons, and parahippocampal gyrus (14). Studies addressing cortical thickness, in turn, have reported thinning in the frontal cortex, lingual gyrus, and fusiform gyrus (12), as well in medial superior frontal, orbitofrontal, anterior cingulate cortices, and dorsolateral primary motor cortex (13).

In the current work, we aimed to investigate [1] cognitive impairment in a sample of IRBD patients compared to healthy controls, [2] MRI gray matter changes and [3] correlation between cognitive impairment and cortical and subcortical atrophy.

## Methods

### Participants

Twenty patients with IRBD without cognitive or motor complaints at the time of diagnosis were recruited from our multidisciplinary sleep unit. Diagnosis of IRBD required a history of dream-enacting behaviors, video-polysomnographic demonstration of REM sleep without atonia and absence of other neurological diseases (15, 16). Twenty-seven healthy subjects without cognitive, motor, or sleep complaints were recruited from the Institut de l'Envelliment (Barcelona, Spain).

Exclusion criteria consisted of: [1] Presence of psychiatric and/or neurologic comorbidity, [2] low global IQ score estimated by the Vocabulary subtest of the Wechsler Adult Intelligence Scale, 3rd edition (scalar score ≤7 points), [3] MMSE score <25, [4] claustrophobia, [5] MRI movement artifacts, and [6] no evidence in HC of sleep disorders or mild cognitive impairment.

The study was approved by the Ethics Committee of the University of Barcelona (IRB00003099) and Hospital Clinic (HCB/2014/0224). All subjects provided written informed consent to participate after full explanation of the procedures involved.

### Neuropsychological and Clinical Assessment

Participants were evaluated with a neuropsychological battery assessing the main cognitive domains impaired in alpha-synuclein-related neurodegenerative diseases. Attention and working memory were assessed with the Trail Making Test (TMT, parts A and B) (in seconds), Digit Span Forward and Backward, the Stroop Color-word Test, and the Symbol Digits Modalities Test (SDMT)-Oral version. Executive functions were evaluated with phonemic (words beginning with the letter “p” in 1 min) and semantic (animals in 1 min) fluencies. Language was assessed by the total number of correct responses in the short version of the Boston Naming Test (BNT). In the memory domain, we assessed total learning recall (sum of correct responses from trial I to trial V), delayed recall (total recall after 20 min), and recognition abilities using Rey's Auditory Verbal Learning Test (RAVLT total, RAVLT recall, and RAVLT recognition, respectively). Visuospatial and visuoperceptual functions were assessed with Benton's Judgement of Line Orientation (JLO), Visual Form Discrimination (VFD), and Facial Recognition (FRT) tests (17). Expected *z* scores adjusted for age, sex, and education for each test and each subject were calculated based on a multiple regression analysis performed in the HC group (18).

Beck Depression Inventory II (19), Starkstein's Apathy Scale (20), and the Neuropsychiatric Inventory (NPI) (21) were used to assess neuropsychiatric symptomatology.

### MRI Acquisition

MRI data were acquired with a 3T scanner (MAGNETOM Trio, Siemens, Germany). The scanning protocol included high-resolution 3-dimensional T1-weighted images acquired in the sagittal plane (TR = 2,300 ms, TE = 2.98 ms, TI = 900 ms, 240 slices, FOV = 256 mm; 1 mm isotropic voxel) and an axial FLAIR sequence (TR = 9,000 ms, TE = 96 ms).

### MRI Preprocessing and Cortical Thickness Analysis

*FreeSurfer* software was used to estimate cortical thickness. This study sample is part of an extensive cohort recruited since 2010, for that reason T1-weighted images were preprocessed with the FreeSurfer 5.1 version (available at https://surfer.nmr.mgh.harvard.edu/ since 2011). The 3D cortical surface model used in this estimation is created using intensity and continuity information, as described in detail by the authors (22). Independent steps are performed in the initial preprocessing of images for each subject: removal of non-brain tissue, automated Talairach transformation, intensity normalization (23), tessellation of the gray matter/white matter boundary, automated topology correction (24), and accurate surface deformation to optimally place the gray matter/white matter and gray matter/cerebrospinal fluid (CSF) boundaries (22). The resulting representation of cortical thickness is calculated as the distance between white and gray matter surfaces at each vertex of the reconstructed cortical mantle (23). In our study, results for each subject were carefully inspected visually to ensure accuracy of registration, skull stripping, segmentation, and cortical surface reconstruction. Cortical thickness maps were smoothed using a circularly symmetric Gaussian kernel across the surface with a full width at half maximum (FWHM) of 15 mm.

Comparisons between groups were assessed using a vertex-by-vertex general linear model introducing age as a covariate (FreeSurfer 5.1). A subsequent analysis introducing both age and sex as covariates was computed. Vertex-wise correlations between cortical thickness and cognitive measures were computed in the IRBD group. In order to avoid clusters appearing significant purely by chance (i.e., false positives), Monte Carlo Null-Z Simulation with 10,000 iterations was applied to cortical thickness maps to provide clusterwise correction for multiple comparisons; results were thresholded at a corrected *p*-value of 0.05 (25).

### Subcortical Segmentation

Automated subcortical segmentation performed with FreeSurfer (version 5.1) was used to estimate subcortical volumetry. Estimated Total Intracranial Volume (eTIV) was obtained to correct volumetric data for inter-individual differences in head sizes.

### Hippocampal Shape Analysis

FIRST, FSL's model-based segmentation/registration tool was used for hippocampal shape analysis (https://fsl.fmrib.ox.ac.uk/fsl/fslwiki/FIRST) (26). Segmentation of both hippocampi with automated boundary correction were generated. We then used first-utils to run a vertex-wise analysis on the results. FSL's Randomize script (27, 28) with 5,000 random permutations of the data was used to study inter-group differences. ETIV was estimated with FSL according to ENIGMA's imaging protocol (http://enigma.usc.edu/). Age and eTIV were introduced as covariates in the analyses. A subsequent analysis introducing both age and sex as covariates was computed. All results were thresholded at *p* < 0.05. Correlations with cognitive variables were performed.

### Segmentation of Hippocampal Subfields

FreeSurfer automated hippocampal subfield segmentation [version 6.0 (29)], was used to estimate individual hippocampal subfield volumes. Segmentation analysis was conducted for the right hippocampus. Since no previous works have studied hippocampal subfields in IRBD patients, an exploratory analysis was undertaken, and results were considered significant if they showed a *p*-value of *p* < 0.05. All hippocampal subfield measures were introduced in a general linear model with eTIV as a covariate.

### Statistical Analyses

Statistical analyses of neuropsychological, demographic, clinical, and MRI volumetric data were carried out using the statistical package SPSS-24 (2016; Armonk, NY: IBM Corp.). Student's *t*-test was used to assess group differences between IRBD and healthy subjects in clinical variables. The general linear model was used to assess group differences in neuropsychological variables and MRI volumetric data. Pearson's chi-squared test was applied to assess group differences in categorical variables. Correlations between structural measures and neuropsychological scores were analyzed using Pearson's correlation. Age, sex, and eTIV were introduced as covariates when needed.

## Results

### Sociodemographic, Clinical, and Neuropsychological Data

Groups did not differ significantly in any demographical measure. The interval between IRBD diagnosis and time of neuroimaging was 3.1 +/– 3.5 years. Inter-group comparisons of clinical, sociodemographic, and neuropsychological variables are shown in Tables 1, 2, respectively.

**Table 1:** Demographic and clinical characteristics.

	**HC (*n* = 27)**	**IRBD (*n* = 20)**	**Test stat/*p*-value**
Age (years)	66.4 (9.9)	71.3 (7.8)	1.83/0.073
Education (years)	12.19 (4.3)	11.9 (4.9)	0.25/0.804
Sex (male/female)	(13/14)	(14/6)	2.24/0.134
Neuropsychiatric Inventory	1.9 (2.5)	6.4 (5.9)	**3.17/0.004**
Beck Depression Inventory II	5.1 (4.7)	7.0 (5.0)	1.30/0.201
Starkstein's Apathy Scale	8.9 (5.3)	10.9 (5.6)	1.20/0.237
Disease duration (years)	–	3.1 (3.5)	–
MDS-UPDRSIII	–	2.4 (1.9)	–

HC, healthy controls; IRBD, idiopathic rapid eye movement sleep behavior disorder; MDS-UPDRSIII, Movement Disorder Society Unified Parkinson's Disease Rating Scale motor section. Group differences between HC and IRBD were tested using Student's t-test. Differences in categorical variables were analyzed with Pearson's chi-squared test. Measures are presented as mean (standard deviation) for continuous variables.

*In bold highlighted those results that reached statistical significance*.

**Table 2:** Group comparison of neuropsychological performance.

	**HC (*n* = 25)**	**IRBD (*n* = 20)**	**Test stat/*p*-value**	**Effect size**
MMSE	29.4 (0.8)	28.2 (1.6)	10.21/0.003^*^	0.95
VFD	29.6 (2.6)	29.2 (3.5)	0.01/0.914	
JLO	24.7 (3.8)	22.8 (5.2)	1.08/0.303	
FRT Short	23.1 (1.9)	21.4 (2.3)	4.96/0.031^*^	0.81
Phonetic fluency	15.8 (4.4)	12.9 (4.8)	3.38/0.073	
Semantic fluency	19.5 (3.2)	15.4 (4.9)	9.24/0.004^*^	0.99
RAVLT total	48.9 (6.8)	41.7 (8.5)	8.44/0.006^*^	0.94
RAVLT recall	10.4 (2.6)	8.0 (3.5)	6.41/0.015^*^	0.78
RAVLT recognition	14.6 (0.8)	13.7 (1.5)	**6.28/0.016**	0.75
Direct Digits	5.2 (1.3)	5.3 (1.5)	0.05/0.827	
Indirect Digits	4.2 (1.1)	4.4 (0.8)	0.44/0.509	
Stroop W	96.6 (14.7)	89.0 (16.8)	1.68/0.202	
Stroop C	64.4 (10.6)	55.9 (11.1)	**5.21/0.028**	0.78
Stroop WC	34.3 (11.3)	29.5 (9.7)	1.18/0.283	
SDMT	48.4 (9.2)	39.1 (12.5)	**6.10/0.018**	0.85
TMTA	36.9 (11.4)	53.9 (23.7)	8.95/0.005^*^	0.91
TMTB	94.1 (49.8)	143.4 (67.5)	5.60/0.023^*^	0.83
BNT	13.9 (0.9)	13.2 (0.9)	5.30/0.026^*^	0.78

BNT, Boston Naming Test; FRT Short, Facial Recognition test short form; HC, healthy controls; IRBD, idiopathic rapid eye movement sleep behavior disorder; JLO, Benton's Judgment of Line Orientation test; MMSE, Mini-mental state examination; RAVLT, Rey's Auditory Verbal Learning Test; RAVLT recall, total recall after 20 min; RAVLT recognition, total recognition after 20 min; RAVLT total, sum of correct responses from trial I to trial V; Symbol Digits Modalities Test (SDMT)-Oral version; Stroop W, Stroop Word; Stroop C, Stroop Color; Stroop WC, Stroop Word-Color; TMTA, Trail Making Test part A; TMTB, Trail Making Test part B; VFD, Visual Form Discrimination. Group differences between HC and IRBD were tested using general linear model with age as a covariate. In bold highlighted those results that reached statistical significance.

* *Comparisons that remain significant (p < 0.05) when age, sex and years of education were regressed out. Cohen's d effect size was computed for significant effects. Measures are presented as mean (standard deviation)*.

### Cortical Thickness Analysis

**Figure 1:**
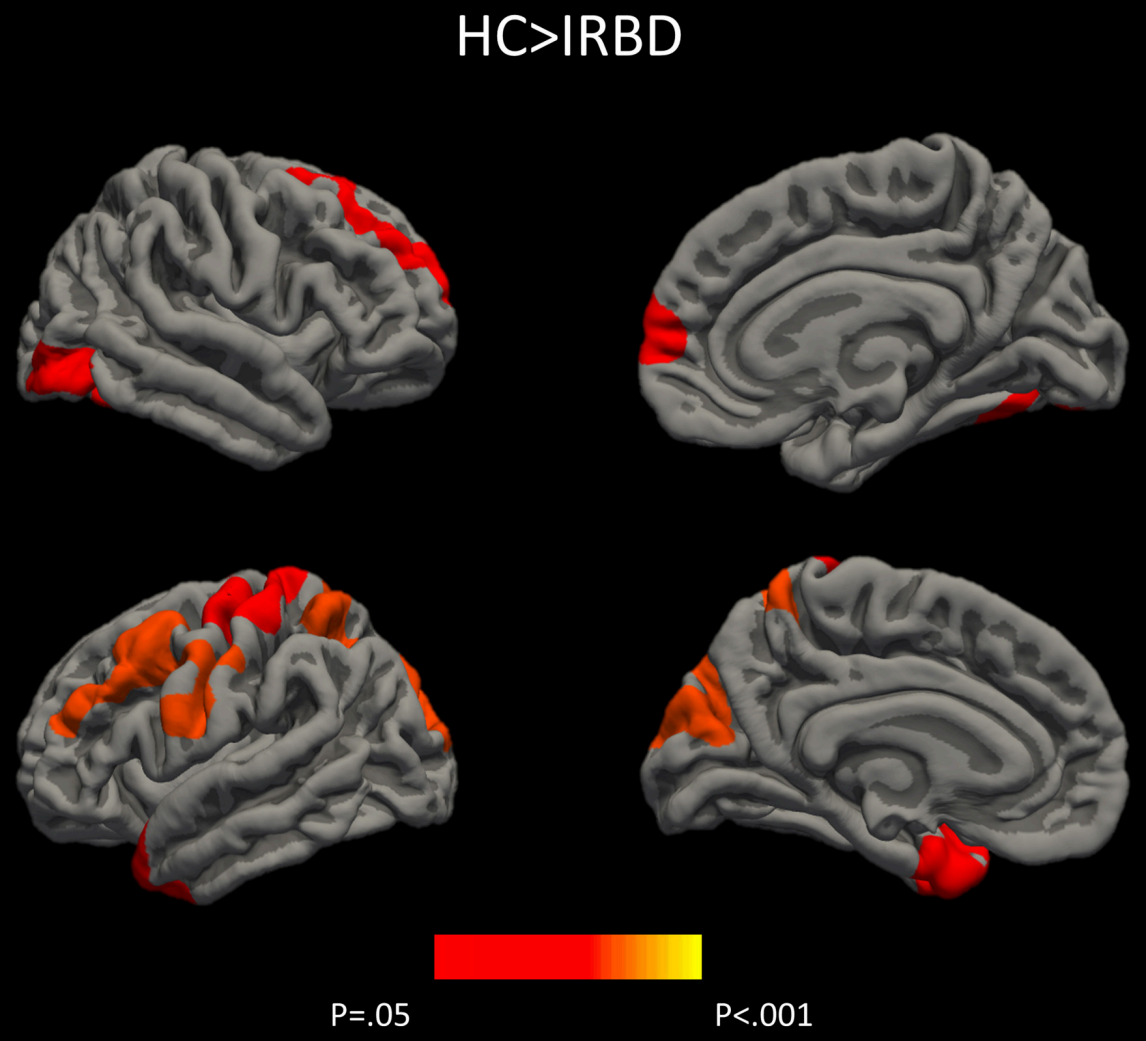
Differences between HC and IRBD patients in cortical thickness. Image show vertex-wise cortical thickness differences between HC and IRBD patients when age was introduced as covariate. Significant clusters are highlighted in warm colors. Results after FWE correction with Monte Carlo simulation and threshold at *p* ≤ 0.05. *Graphics program:* Freeview from FreeSurfer (https://surfer.nmr.mgh.harvard.edu/fswiki/FreeviewGuide), and edited with Microsoft PowerPoint®.

Results of whole-brain cortical thickness analyses showed that IRBD patients had cortical thinning compared with HC in left superior parietal, post-central, and fusiform regions, as well as in right superior frontal and lateral occipital regions. Left superior parietal, left post-central, and right lingual and paracentral regions remained significant after controlling for both, age and sex Figure 1, Table 3, and Supplementary Material 1.

**Table 3:** Differences between HC and IRBD patients in cortical thickness.

	**Cluster size (mm^**2**^)**	**MNI305 space**			**Clusterwise *p*-value**	**Cluster anatomical annotation**
		**X**	**Y**	**Z**		
**HC>RBD (AGE AS COVARIATE)**						
**LH Clusters**						
1	4846.8	−22.9	−74.4	29.9	< 0.001	Superior parietal
2	2935.7	−52.9	−11.7	23.8	< 0.001	Post-central
3	1987.3	−24.1	−31.1	63.3	0.003	Post-central
4	1438.7	−34.0	2.0	−39.4	0.041	Fusiform
**RH Clusters**						
1	2272.63	21.3	28.4	39.9	0.003	Superior frontal
2	1774.5	43.5	−78.7	−6.4	0.017	Lateral occipital
**HC>RBD (AGE AND SEX AS COVARIATES)**						
**LH Clusters**						
1	2863.5	−23.1	−73.5	30.0	< 0.001	Superior parietal
2	2672.3	−49.8	−10.3	23.2	< 0.001	Post-central
3	1767.1	−32.3	−54.8	61.5	0.007	Superior parietal
**RH Clusters**						
1	2195.6	9.1	−69.1	−1.1	0.003	Lingual
2	1530.3	8.6	−8.5	66.6	0.045	Paracentral

*HC, healthy controls; IRBD, idiopathic rapid eye movement sleep behavior disorder; LH, left hemisphere; RH, right hemisphere. Results after FWE correction with Monte Carlo simulation and threshold at p ≤ 0.05*.

### Subcortical Segmentations

Inter-group analysis of subcortical segmentations showed IRBD patients had reduced hippocampal volume (*F* = 7.730; *P* = 0.008; Cohen's *d* effect size = 0.77). In a subsequent analysis right hippocampal volume achieved significance (*F* = 5.086; *P* = 0.029; Cohen's *d* effect size = 0.85), whereas no differences were found for the left hippocampus (*F* = 2.491; *P* = 0.122). These results remained significant after controlling for both, age and sex Table 4.

**Table 4:** Deep gray matter measures (mm^3^).

	**HC (*n* = 27)**	**RBD (*n* = 20)**	**Test stat/*p*-value**	**Effect size**
Thalamus	12707.1 (1319.2)	12413.8 (1327.6)	1.325/0.256	
Caudate	6609.0 (962.9)	6505.5 (1095.2)	0.337/0.564	
Putamen	9682.2 (1051.0)	9213.5 (1201.5)	2.359/0.132	
Pallidum	3062.9 (363.3)	2968.7 (378.5)	1.208/0.278	
Amygdala	3198.9 (521.1)	2990.1 (431.3)	2.855/0.098	
Accumbens	1051.1 (186.1)	975.5 (182.5)	1.938/0.171	
Brain stem	20318.7 (2455.7)	20364.2 (2270.1)	0.057/0.813	
Hippocampus	8137.0 (941.7)	7447.5 (840.3)	7.730/0.008^*^	0.77
Right	4082.4 (482.0)	3709.1 (397.3)	5.087/0.029^*^	0.85
Left	4054.6 (479.9)	3738.4 (479.6)	2.491/0.122	

HC, healthy controls; IRBD, idiopathic rapid eye movement sleep behavior disorder. Measures are presented as means (standard deviation). In bold highlighted those results that reached statistical significance.

* *Comparisons that were significant (p < 0.05) when estimated Total Intracranial Volume, age and sex were introduced as covariates, Cohen's d effect size was computed for significant effects*.

### Hippocampal Shape Analysis

**Figure 2:**
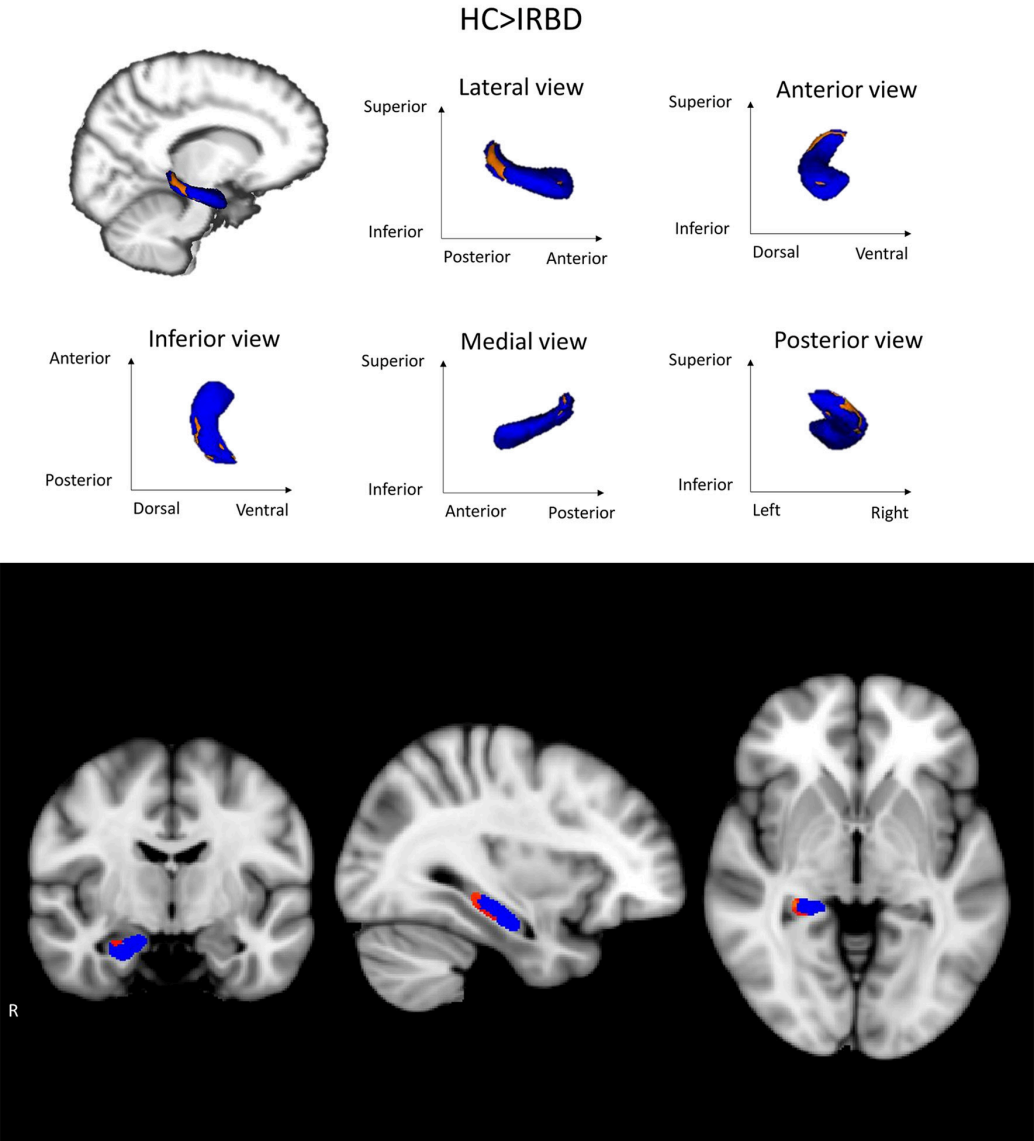
Hippocampal shape differences between HC and IRBD patients. Images show right hippocampus shape differences between HC and IRBD patients when age and estimated Total Intracranial Volume were introduced as covariates. Significant clusters of shape differences (local atrophy) between the groups are highlighted in warm colors (corrected *P* < 0.05). Results are overlaid on the right hippocampus mask (blue) and displayed over the sagittal, coronal and axial sections of the MNI standard brain. *Graphics Program:* fslview from FSL (https://fsl.fmrib.ox.ac.uk/fsl/fslwiki/FslView/UserGuide) and edited with Microsoft PowerPoint®.

Shape analysis showed inter-group differences for the right hippocampus in a cluster located in its posterior region, including mainly the CA1, the hippocampal tail, the subiculum, and the dentate gyrus Figure 2. A clear tendency to significance was seen when sex was introduced as a covariate (*P* = 0.058).

### Segmentation of Hippocampal Subfields

An exploratory analysis of right hippocampal subfields showed that, in comparison with the HC group, IRBD patients had a significant reduction in the *right cornu ammonis 1* (CA1) (*F* = 5.685; *P* = 0.021; Cohen's *d* effect size = 0.616), *molecular layer* (*F* = 5.764; *P* = 0.021; Cohen's *d* effect size = 0.645)*, granule cell layer of the dentate gyrus* (*F* = 4.863; *P* = 0.033; Cohen's *d* effect size = 0.579)*, and cornu ammonis 4* (CA4) (*F* = 4.890; *P* = 0.032; Cohen's *d* effect size = 0.586) Supplementary Material 2.

### Correlation Analyses

To search for cortical substrates of cognitive changes in patients, we performed cortical thickness analyses. Nevertheless, none cognitive function correlated significantly with cortical thickness maps. Moreover, no significant correlations in the IRBD group were found between measures of hippocampal atrophy and either memory scores or other cognitive variables.

## Discussion

To our knowledge this is the first study that combine MRI structural data and cognitive assessment to compare IRBD patients and a group of healthy controls. Our results showed that IRBD patients had posterior based cognitive impairment, cortical atrophy and reduction of the hippocampal volume. In this study we explore the hippocampal structure through different structural MRI techniques and found reductions in right whole-hippocampus volume, mainly in posterior regions.

Similarly to previous neuropsychological results on IRBD, in the current study, patients differed from controls in several cognitive domains (8), namely attention (30, 31), verbal memory (30–33), executive function (30, 34, 35) and semantic fluency (30, 31, 36). Our results showed large effect size for facial recognition, semantic fluency, RAVLT total learning, SDMT, TMTA, and TMTB tests. Interestingly, a previous work found TMT, verbal fluency and Stroop Color Word test, were the best predictors of Lewy bodies dementia in IRBD patients (9). As far as we know, this is the first work assessing facial recognition in IRBD patients and showing lower scores in comparison to HC. In this sense, there are previous literature identifying facial emotion recognition (37) and facial recognition impairment in PD patients (38) suggesting this posterior-based dysfunction could be a cognitive biomarker of PD conversion in IRBD patients. Further longitudinal studies are needed to elucidate this issue.

Cortical thickness analysis showed in comparison to HC, IRBD patients had atrophy in left superior parietal, post-central, and fusiform regions, as well as in right superior frontal and lateral occipital regions. When age and sex were introduced as covariates, left superior parietal, left post-central, and right lingual and paracentral regions remained significant. These results agree with those obtained by Rahayel et al. (12, 13) regarding the involvement of dorsolateral prefrontal and occipito-medial regions. In addition, we observed superior parietal thinning similarly to that described in non-demented PD (39, 40). Interestingly, similar to our results fusiform gyrus and parietal lobe thinning was observed in cross sectional (41, 42) and longitudinal DLB dementia studies (43).

Deep gray matter analysis showed a reduction in the right hippocampus in IRBD patients, there are some works indicating the existence of medial temporal lobe abnormalities in such patients using other neuroimaging approaches. For example, studies using whole-brain VBM approach, reported increased hippocampal gray matter density (10), but also gray matter reduction in related structures such as the parahippocampal gyrus (14). On the other hand, brain perfusion studies have found an increased metabolic activity of the hippocampus in IRBD patients (30, 44–47). Some of these works have found this effect specifically in the right hippocampus (30, 44). Furthermore, hippocampal perfusion in IRBD has been reported as a predictor of PD or DLB evolution (45).

Exploratory analyses indicated a trend to reduction in IRBD patients in the right CA1 and CA4 subfields, right molecular layer, and right granule cell layer of the dentate gyrus. In agreement with our findings, reductions in the CA1 (48, 49), CA4-DG (50), subiculum, and presubiculum (48) have been found in DLB patients. By contrast, a previous work reported preservation of hippocampal subfields in DLB patients (51). Neuropathological studies in DLB evidenced greater Lewy pathology in the CA2 (52), but also in the entorhinal cortex, CA1, CA3, CA4, and the subiculum (53).

In a longitudinal study including a larger sample of MCI subjects, the hippocampal volume reduction was reported as predictor of evolution to AD rather than DLB (54). In the same line, AD showed higher hippocampal atrophy than PDD and healthy controls, and regional vulnerability differed between AD and PDD specifically in whole right hippocampus and right subiculum (55). Previous literature have found the progression of IRBD patients is mainly to alpha-synucleinopathies such as DLB, PD, and MSA (3, 4), and not to AD. Further recent findings also provide evidence that APOE-ε4 is linked to hippocampal atrophy and learning/memory phenotypes across the AD/DLB spectrum (56). However, in light of previous data, we cannot rule out that some of our patients could evolve to AD, in this sense could be interesting to obtain APOE ε4 genotypic profile.

Finally, it can be difficult to dissociate if hippocampal atrophy and memory impairment are due to sleep problems itself or to sleep problems plus the neurodegenerative process. Sleep deprivation has been related to hippocampal dysfunction and volume reduction (57), so we cannot attribute our results to the neurodegenerative process *per se*. For that reason, studies including other sleep disturbances as control group are needed.

Contrary to our hypothesis, we did not find significant correlations between cognitive performance and brain atrophy. However, there is coherence between the detected brain atrophy by MRI and neuropsychological profile observed in our sample. This could be due to the small sample size, or the lack of linear relationship between structural changes and neuropsychological impairment. A previous work studying IRBD patients reported that cortical thinning was associated with lower performance in cognitive domains, namely attention and executive functions, learning and memory, and visuospatial abilities (58). In this setting, right hippocampus volume has been related to spatial memory abilities (59) and spatial mapping (60), with a greater role of posterior hippocampi (60). The lack of association between hippocampal volume and memory performance in our study might be improved in the future by using an extensive neuropsychological assessment including visual and spatial memory tests, as well as test paradigms such as the Free and Cued selective Reminding Test to study memory dissociations between recall and recognition in IRBD patients.

Despite the novel findings described above, some limitations of the current study should be acknowledged. First, the relatively small sample size requires caution in generalizing our results, therefore these findings need to be reproduced in larger samples. Second, considering the exploratory nature of the hippocampal subfield analysis, we did not apply correction for multiple comparisons

In conclusion, we found reductions of mainly posterior cortical thickness and right hippocampal volume in IRBD, alongside evidence of cognitive impairment. This pattern is similar to cognitive decline and atrophy observed in PD and DLB.

## Author Contributions

CJ contributed in the design of the study. AC, AA, and CU contributed to the analysis of the data and AC, BS, CJ, MS, AA, CU, HB, CG, JS, YC, and AI contributed to the interpretation of the data. AC and CJ contributed to the draft of the article. AC, BS, CJ, MS, AA, CU, HB, CG, JS, YC, NB, and AI revised the manuscript critically for important intellectual content and approved the final version of the manuscript.

### Conflict of Interest Statement

The authors declare that the research was conducted in the absence of any commercial or financial relationships that could be construed as a potential conflict of interest.
