# Supplementary material for: Cortical Gray Matter and Hippocampal Atrophy in Idiopathic Rapid Eye Movement Sleep Behavior Disorder
Source: Front Neurol. 2019 Apr 5;10:312. doi: 10.3389/fneur.2019.00312 (PMC6459930; doi:10.3389/fneur.2019.00312)
Supplement: Supplementary file 1 [file Table_1.pdf]

Supplementary Material

Cortical gray matter and hippocampal atrophy in idiopathic Rapid Eye Movement sleep behavior disorder

A. Campabadal; B. Segura; C. Junque*; M. Serradell; A. Abos; C. Uribe; H.C. Baggio; C. Gaig; J. Santamaria; Y Compta; N. Bargallo; A. Iranzo

***Correspondence:** Prof. Carme Junque.
Medical Psychology Unit, Department of Medicine. University of Barcelona
Casanova 143 (08036) Barcelona, Spain
Phone: (+34) 93 402 45 70 // Fax: (+34) 93 403 52 94 // E-mail: cjunque@ub.edu

Supplementary Material 1.

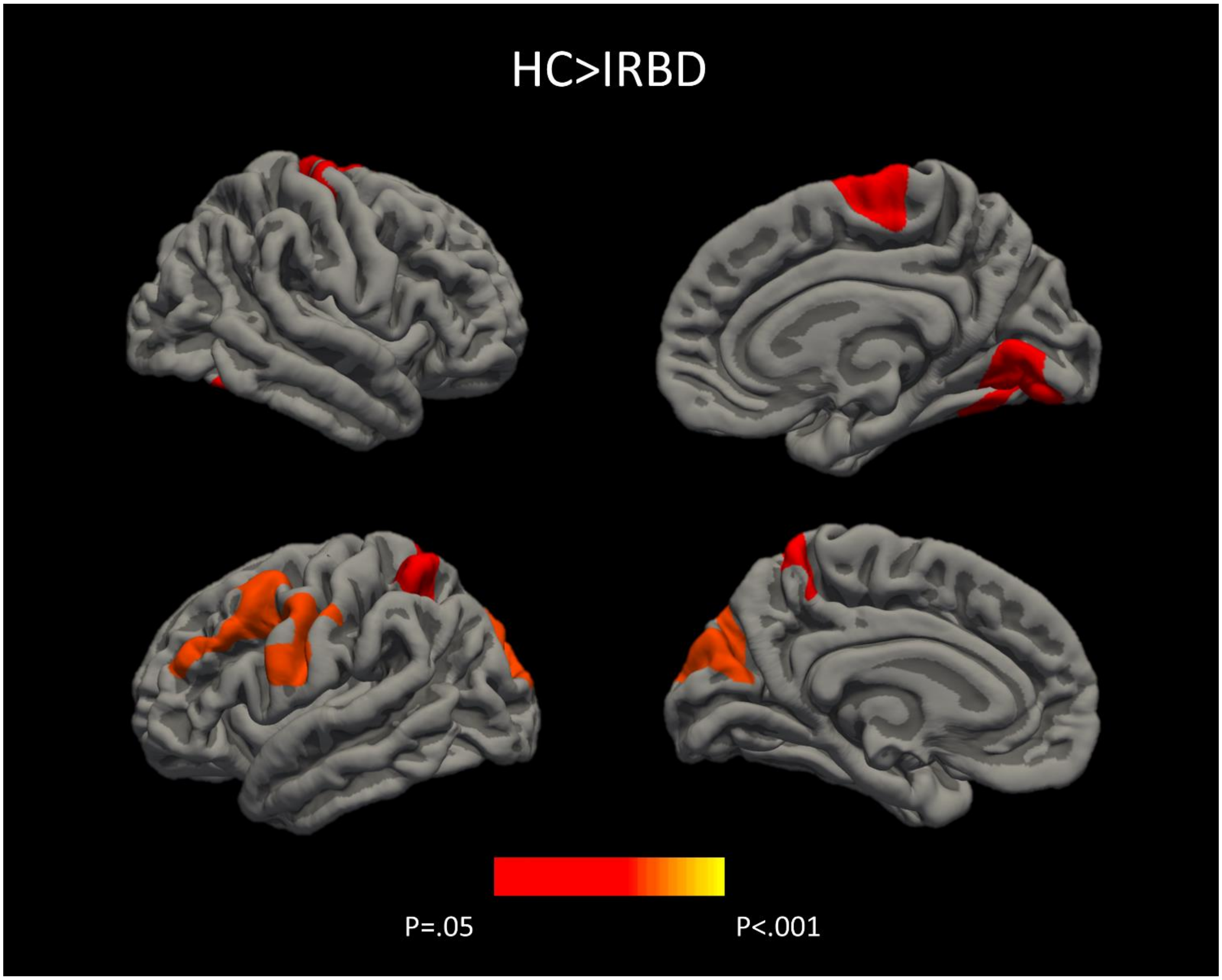

Images show vertex-wise cortical thickness differences between HC and IRBD patients when age and sex were introduced as covariates. Significant clusters are highlighted in warm colors. Results after FWE correction with Monte Carlo simulation and threshold at $p \leq 0.05$.

Graphics program: Freeview from FreeSurfer (https://surfer.nmr.mgh.harvard.edu/fswiki/FreeviewGuide) and edited with Microsoft PowerPoint®.

Supplementary Material 2. Hippocampal subfield volumes (mm^3)

	**HC (*n*=27)**	**IRBD (*n*=20)**	**Test stat/*p*-value**	**Effect size**
**Right Hippocampal tail**	514.4 (85.9)	475.9 (54.3)	*3.819/0.057*	
**Right subiculum**	429.1 (58.1)	400.0 (59.9)	*3.316/0.075*	
**Right CA1**	641.0 (79.3)	594.0 (73.2)	**5.685/0.021**	0.616
**Right hippocampal fissure**	170.2 (25.5)	173.1 (26.9)	0.098/0.756	
**Right presubiculum**	289.1 (47.8)	276.7 (46.6)	1.071/0.306	
**Right parasubiculum**	60.8 (16.1)	65.0 (15.7)	0.665/0.419	
**Right molecular layer HP**	570.7 (73.9)	524.7 (68.6)	**5.764/0.021**	0.645
**Right granule cell of dentate gyrus**	305.8 (41.1)	282.9 (37.8)	**4.863/0.033**	0.579
**Right CA2-CA3**	227.1 (33.6)	211.2 (33.1)	3.025/0.089	
**Right CA4**	264.8 (33.5)	246.3 (29.5)	**4.890/0.032**	0.586
**Right fimbria**	78.2 (25.7)	67.6 (27.2)	2.033/0.161	
**Right HATA**	59.8 (9.1)	56.4 (10.4)	1.951/0.169	

Abbreviations: HC, healthy controls; IRBD, idiopathic rapid eye movement sleep behavior disorder. General lineal model with estimated Total Intracranial Volume as a covariate was used, Cohen's *d* effect size was computed for significant effects. Measures are presented as means (standard deviation). In bold highlighted those results that reached statistical significance.
